# Adaptive sensitivity-fisher regularization for heterogeneous transfer learning of vascular segmentation in laparoscopic videos

**DOI:** 10.1007/s11548-025-03404-2

**Published:** 2025-06-06

**Authors:** Xinkai Zhao, Yuichiro Hayashi, Masahiro Oda, Takayuki Kitasaka, Kazunari Misawa, Kensaku Mori

**Affiliations:** 1 Graduate School of Informatics, Nagoya University, Furo-cho, Chikusaku, Nagoya, Aichi, Japan; 2 Information Technology Center, Nagoya University, Furo-cho, Chikusaku, Nagoya, Aichi, Japan; 3 School of Information Science, Aichi Institute of Technology, 1247 Yachigusa, Yagasa-cho, Toyota, Aichi, Japan; 4 Aichi Cancer Center, 1-1 Kanokoden, Chikusa-ku, Nagoya, Aichi, Japan; 5 Research Center for Medical Bigdata, National Institute of Informatics, 2-1-2 Hitotsubashi, Chiyoda-ku, Tokyo, Japan

**Keywords:** Laparoscopic surgery, Vascular segmentation, Transfer learning

## Abstract

**Purpose:**

This study aims to enhance surgical safety by developing a method for vascular segmentation in laparoscopic surgery videos with limited visibility. We introduce an adaptive sensitivity-fisher regularization (ASFR) approach to adapt neural networks, initially trained on non-medical datasets, for vascular segmentation in laparoscopic videos.

**Methods:**

Our approach utilizes heterogeneous transfer learning by integrating fisher information and sensitivity analysis to mitigate catastrophic forgetting and overfitting caused by limited annotated data in laparoscopic videos. We calculate fisher information to identify and preserve critical model parameters while using sensitivity measures to guide adjustment for new task.

**Results:**

The fine-tuned models demonstrated high accuracy in vascular segmentation across various complex video sequences, including those with obscured vessels. For both invisible and visible vessels, our method achieved an average Dice score of 41.3. In addition to outperforming traditional transfer learning approaches, our method exhibited strong adaptability across multiple advanced video segmentation architectures.

**Conclusion:**

This study introduces a novel heterogeneous transfer learning approach, ASFR, which significantly enhances the precision of vascular segmentation in laparoscopic videos. ASFR effectively addresses critical challenges in surgical image analysis and paves the way for broader applications in laparoscopic surgery, promising improved patient outcomes and increased surgical efficiency.

**Supplementary Information:**

The online version contains supplementary material available at 10.1007/s11548-025-03404-2.

## Introduction

Accurate vascular localization in laparoscopic surgery is crucial, as it directly impacts surgical outcomes and patient safety [1]. However, in complex laparoscopic scenarios, vessels are often obscured by surrounding tissues, such as fat, making it challenging to locate them accurately [2]. Traditionally, surgeons rely on indocyanine green (ICG) near-infrared fluorescent dyes [3] or Doppler ultrasound [4] to enhance the visibility of obscured vessels. Despite their utility, these methods have several limitations. For example, the Da Vinci^®^ Firefly™ imaging system [5] requires frequent switching between white light and fluorescence imaging, which complicates surgical workflows, while ICG injections carry risks such as allergic reactions and cardiovascular side effects [6]. To overcome these limitations, we aim to develop a method that allows for continuous segmentation of both visible and obscured vessels in laparoscopic videos, using only conventional white light imaging. This approach eliminates the reliance on additional imaging modalities, simplifying surgical workflows and improving safety.

A major challenge in achieving this goal is the lack of annotated laparoscopic vascular datasets for training deep learning models. Annotating vascular locations in surgical videos is a tedious and expertize-intensive process, making it difficult to obtain sufficient training data. To address this issue, we adopt a heterogeneous transfer learning approach. This method enables the transfer of knowledge from large, non-medical datasets to the medical domain [7], reducing the need for annotated surgical datasets while leveraging the diversity and scale of non-medical data to improve performance.

Heterogeneous transfer learning involves transferring knowledge across domains with different feature spaces, data distributions, and label semantics [8]. While this approach has been widely explored in single image tasks [9, 10], its application to video segmentation is less straightforward due to the complexity of modern video segmentation networks, such as STCN [11] and XMem [12]. These networks include components such as memory banks and decoders, which are critical for temporal consistency but are often treated equally during transfer learning, leading to suboptimal adaptation.

To address these limitations, we propose an Adaptive Sensitivity-Fisher Regularization (ASFR) method. Our method evaluates the Fisher information of parameters from pretrained models to identify those critical for maintaining the original task’s performance. It then uses sensitivity analysis to determine which parameters are most responsive to the target laparoscopic dataset. By combining these two measures, ASFR mitigates catastrophic forgetting [13] while optimizing model adaptation for laparoscopic vascular segmentation.

The contributions of this paper are as follows:

- We tackle a novel and challenging task of vascular segmentation in laparoscopic videos, focusing on both visible and obscured vessels, using only white light imaging.
- To address the lack of annotated laparoscopic datasets, we propose a heterogeneous transfer learning framework and introduce an ASFR method to bridge the domain gap.
- We demonstrate the effectiveness of our approach across diverse video segmentation architectures, achieving robust performance in segmenting vascular structures in laparoscopic videos.

## Related works

### Vascular recognition for laparoscopic surgery

In laparoscopic sleeve gastrectomy, the ligation of short gastric vessels is a critical preparatory step for subsequent operations [14]. One of the most frequent and significant errors during this process is bleeding, which requires heightened attention from surgeons [15, 16]. Therefore, it is crucial to accurately and continuously locate gastric vessels before ligation. While gastric vessels can be localized preoperatively using CT imaging or ICG dye [3, 17], these methods do not address the intraoperative challenge of locating vascular structures directly within laparoscopic videos.

Most existing studies on laparoscopic segmentation focus on static images, such as anatomical structures or surgical instruments [18, 19], rather than dynamic video data. However, the need for continuous and precise vascular localization in laparoscopic videos remains an open and clinically significant challenge. In this work, we address this gap by proposing a novel approach to vascular localization, which is designed to enhance both the clinical relevance and technical robustness of vascular segmentation in laparoscopic videos.

### Heterogeneous transfer learning

Lack of training data and annotations is a common problem in the field of medical image processing [20, 21]. To address the lack of vascular annotations in laparoscopic videos, we adopt heterogeneous transfer learning, which enables knowledge transfer across domains with differing feature spaces, data distributions, and label semantics [7, 8]. The success of transfer learning hinges on how to appropriately select prior knowledge to transfer to new tasks, which faces a dilemma between catastrophic forgetting and negative transfer [22, 23]. TERD [24], NTMEL [25], IR [26] address these issues by designing different regularization methods. While effective in image tasks, its application to video segmentation remains challenging due to the complexity of models like STCN [11] and XMem [12], which include diverse components such as memory banks and decoders.

Fisher information is widely used to measure parameter importance and prevent catastrophic forgetting in transfer learning [9, 27]. Recent studies reveal universal statistical properties of the Fisher Information Matrix, highlighting its potential in guiding parameter adaptation [28]. To overcome these challenges, we propose an ASFR method. By combining Fisher information with sensitivity analysis, our method effectively identifies and prioritizes parameters critical for robust vascular segmentation in laparoscopic videos.

## Method

In this section, we first clarify the problem definition, then introduce our proposed ASFR method, and finally describe the process of fine-tuning the network using the proposed method.

### Problem definition

**Fig. 1:**
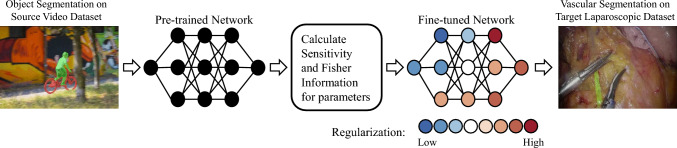
This paper aims to adapt a network originally pretrained on non-medical image video segmentation datasets for the task of vascular segmentation in laparoscopic videos. To preserve valuable information from the pretrained network and filter out non-essential information, we propose a method that uses sensitivity and Fisher information to guide regularization during fine-tuning

**Fig. 2:**
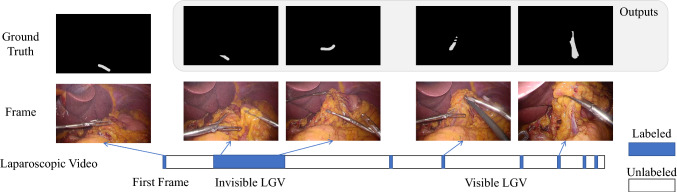
The task requires using the annotations from the first frame as input to the segmentation network, which then outputs the location of the LGV in each subsequent frame. Additionally, some frames used for training and evaluating the model are not consecutive, which allows for the assessment over extended time periods, offering a comprehensive evaluation of segmentation performance across various scene

Our study focuses on transfer learning for vascular segmentation in laparoscopic videos, as shown in Fig. 1. The process involves two datasets: $\textcircled {1}$ **Source Video Dataset**: This is a large dataset of non-medical scenes used for pretraining. Let the input images from the source dataset be denoted by $\varvec{\mathcal {X}} = \{\varvec{X}_1, \varvec{X}_2, \ldots , \varvec{X}_N\}$, where $\varvec{X}_i$ represents an image, and the corresponding labels be denoted by $\varvec{\mathcal {Y}} = \{\varvec{Y}_1, \varvec{Y}_2, \ldots , \varvec{Y}_N\}$, where $\varvec{Y}_i$ represents the label for $\varvec{X}_i$. $\textcircled {2}$ **Target Laparoscopic Dataset**: This dataset is specific to the video segmentation of the Left Gastric Vein (LGV) in long-term laparoscopic surgery videos, as shown in Fig. 2. Let the input video be denoted by $\varvec{\mathcal {V}} = \{\varvec{V}_1, \varvec{V}_2, \ldots , \varvec{V}_T\}$, where $\varvec{V}_t$ represents the frame at time $t$. The initial annotation for the vessel in the first frame is given by $\varvec{A}_1$. The goal is to output segmentation masks $\{\varvec{A}_2, \varvec{A}_3, \ldots , \varvec{A}_T\}$ for the remaining frames.

The segmentation model outputs a predicted mask $\hat{\varvec{A}}_t$ for each frame $\varvec{V}_t$, which can be represented as:

1 $$\begin{aligned} \hat{\varvec{A}}_t, \varvec{M}_t = \mathcal {F}(\varvec{V}_t, \varvec{M}_{t-1}; \varvec{\theta }), \end{aligned}$$

where $\mathcal {F}$ is the segmentation function parameterized by the weights $\varvec{\theta }$, $\varvec{M}_{t-1}$ and $\varvec{M}_{t}$ are the feature memories from the previous frames and for next frame.

Given the challenges posed by limited training data, we utilize STCN [11] and XMem [12], which are trained on the YoutubeVOS [29] and DAVIS [30] datasets. To adapt a model pretrained on the source dataset to the target task of vascular segmentation, we propose a novel heterogeneous transfer learning method.

### Adaptive sensitivity-fisher regularization

To address the limited annotated data, we integrate Fisher Information and sensitivity measures into the transfer learning process. This method involves computing both the Fisher Information matrix and sensitivity measures to guide the regularization process during fine-tuning.

#### Fisher information

Fisher Information quantifies the information of each model parameter contributes to the output predictions, which is crucial when adapting models to new tasks with limited data. It identifies essential parameters to maintain performance on the source task and prevents significant changes that might lead to catastrophic forgetting [9].

Practically, Fisher information matrix $\varvec{F}$ quantifies each network parameter’s importance with respect to the source task. Follow previous works [31, 32], to reduce computation complexity, we consider the diagonal of Fisher information matrix, which is calculated as the expected value of the squared gradient of the log likelihood with respect to the parameter:

2 $$\begin{aligned} \varvec{F}_i = \mathbb {E}\left[ \frac{\partial ^{2} }{\partial (\varvec{\theta }_i^S)^{2}} \mathcal {L}(\varvec{\varvec{\mathcal {X}}|\theta }^S) \right] \approx \mathbb {E}\left[ \left( \frac{\partial \mathcal {L}(\varvec{\varvec{\mathcal {X}}|\theta }^S)}{\partial \varvec{\theta }_i^S}\right) ^2\right] , \end{aligned}$$

where $\mathcal {L}(\varvec{\varvec{\mathcal {X}}|\theta }^S)$ is the log likelihood function of the model parameter $\varvec{\theta }^S$ in source dataset $\varvec{\mathcal {X}}$, which is equivalent to computing the loss of segmentation result, and $\varvec{\theta }_i^S$ is the i-th parameter. And the expected value for the dataset is denoted by $\mathbb {E}[\cdot ]$. This equation computes with respect to the parameters across all data points. The measurement indicates how sensitive the output distribution is to changes in parameters, emphasizing the importance of each parameter in managing transitions between different datasets.

#### Sensitivity measures

Sensitivity measures gauge the robustness of the model’s predictions when exposed to minor perturbations in the input. This aspect is particularly crucial in environments like laparoscopic surgery, where slight changes in video frames can significantly affect the model’s accuracy. High sensitivity indicates a potential risk of overfitting, which is particularly concerning when adapting the model to a small laparoscopic dataset.

For the target vascular dataset, we define sensitivity $\varvec{S}$ as follows:

3 $$\begin{aligned} \varvec{S}_i = \mathbb {E}\left[ \frac{\partial }{\partial \varvec{\theta }_i^S} \left\| \mathcal {F}(\varvec{V}_t + \varvec{\delta }, \varvec{\theta }^S) - \mathcal {F}(\varvec{V}_t, \varvec{\theta }^S) \right\| ^2 \right] , \end{aligned}$$

where $\varvec{V}_t$ represents a frame at time $t$ from the laparoscopic video, $\varvec{\delta } \sim \mathcal {N}(\textbf{0}, \textbf{I})$ denotes Gaussian noise added to the frame, and $\mathcal {F}(\cdot , \varvec{\theta }^S)$ is the video segmentation network performed on a frame with parameters $\varvec{\theta }^S$. This calculation assesses how the addition of noise to each frame impacts the predictive stability of the segmentation model across all subsequent frames. This measure helps ensure that our model remains stable and reliable under the dynamic conditions of surgical video analysis.

#### Fine-tuning and optimization

Fine-tuning for the target task involves minimizing the total loss, which includes both the empirical loss on the new task and a regularization term. This regularization is necessary to ensure the model maintains useful knowledge from its previous training and avoids catastrophic forgetting. By including a penalty term that constrains large deviations from the original parameters, the model balances adapting to the new task with retaining prior knowledge.

The total loss function $\mathcal {L}(\varvec{\theta })$ for fine-tuning is defined as:

4 $$\begin{aligned} \mathcal {L}(\varvec{\theta }) = \mathcal {L}_{\text {target}}(\varvec{\theta }) + \lambda \sum _{i} \left( \varvec{F}_i + \varvec{S}_i\right) (\varvec{\theta }_i - \varvec{\theta }_{i}^{S})^2 \end{aligned}$$

where $\mathcal {L}_{\text {target}}(\varvec{\theta })$ represents the empirical loss on the target task, while $\lambda$ is a regularization parameter that balances the new task’s loss with the preservation of knowledge from the source task. $i$ indexes all parameters. The terms $\varvec{F}_i$ and $\varvec{S}_i$ refer to the Fisher Information and Sensitivity measures, as described in the previous subsections. This combination ensures the model adapts to the new task without losing critical information from its prior training.

### Training procedure

The fine-tuning process consists of the following steps:

1. **Pretraining:** Initially train the model on a vast dataset of non-medical scenes to develop a robust base model ($\varvec{W}_s$).
2. **Fisher Information Calculation:** Compute the Fisher Information matrix using the source dataset $\{\varvec{X}, \varvec{Y}\}$ to identify key parameters.
3. **Sensitivity Calculation:** Assess model sensitivity using the target vascular dataset $\{\varvec{V}, \varvec{A}\}$, crucial for tuning the model’s response to input variations.
4. **ASFR Fine-tuning:** Optimize the model on the target task with a regularized loss function (Eq. 4), balancing new task demands with knowledge preservation.

## Experiments and results

In this section, we delineate the experimental setup and methodology employed to assess the efficacy of our proposed ASFR method. We conduct both qualitative and quantitative comparisons between ASFR and established methods, including Elastic Weight Consolidation (EWC) [9], as well as advanced transfer learning techniques such as Structure Learning with Similarity Preserving (L2SP) [10] and Batch Spectral Shrinkage (BSS) [22].

### Dataset

For the validation and training of our ASFR approach, we utilized an in-house dataset consisting of 22 laparoscopic gastrectomy videos from the Aichi Cancer Center, Japan. The dataset includes annotations for both visible and invisible LGV, with a total of 1,581 frames annotated for visible LGV and 3,444 frames for invisible LGV. To ensure robust evaluation, we partitioned the dataset into distinct sets: 1,225 frames from 4 videos were designated as the test set, 587 frames from 2 videos formed the validation set, and the remaining frames from 18 videos were allocated to the training set. This partitioning ensures comprehensive coverage of variations in LGV visibility and the complexity inherent in surgical procedures.

### Experimental details

The experiments were implemented on the STCN [11] and XMem [12] networks, utilizing their pretrained weights to establish a strong foundational model. Fine-tuning was conducted on a single NVIDIA Tesla V100 GPU. The training protocol involved processing batches of randomly cropped images measuring $320 \times 320$ pixels across 25,000 iterations, with each batch containing 4 images. The hyperparameter $\lambda$ was strategically set to 0.5 to balance the dual objectives of minimizing the new task’s loss and preserving the fidelity of previously acquired knowledge.

### Performance metrics

Since our task involves segmenting the LGV as it transitions from invisible to visible states, we evaluate our method and existing methods using the Dice score in two parts: the segmentation of invisible LGV and the segmentation of visible LGV. To provide an overall measure of performance, we also compute the average of these two Dice scores.

It is important to note that accurately determining the boundaries of the LGV in laparoscopic images presents challenges, particularly when vessels are partially occluded. As such, the ground truth annotations may not be entirely precise. Therefore, while the Dice score provides a useful metric for comparing segmentation performance, it should be considered as a reference and may not precisely reflect the actual segmentation accuracy in numerical terms, especially for invisible vessels.

### Quantitative comparison

We evaluated our method and previous approaches using 4 lengthy laparoscopic videos, each averaging 6 min and 46 s (10,162.5 frames). The results, presented in Table 1. All methods exhibit relatively large standard deviations, which reflect the Dice score variability from frame to frame, due to the inherent ambiguity in vascular location. Our method outperformed others on two different network architectures, both in segments immediately following the labeled first frame (invisible vessels) and in later frames (visible vessels). This differentiation in performance underscores the pretrained network’s transferability in video segmentation and its adaptability to laparoscopic video.

**Table 1:** Comparison of Dice scores of different transfer learning methods on STCN [11] and XMem [12]. P indicates the use of pretrained weights, and R indicates the use of regularization. EWC [9] uses only Fisher Information (Sect. 3.2.1) and serves as an ablation study. Highest and second highest results are highlighted in bold and underlined

P	R	Method	STCN			XMem		
			Invisible	Visible	Average	Invisible	Visible	Average
–	–	Baseline	2.0$\,\,\pm \,\, 8.9$	25.2$\,\,\pm \,\, 31.8$	11.9$\,\,\pm \,\, 25.1$	4.9$\,\,\pm \,\, 15.5$	34.8$\,\,\pm \,\, 35.1$	17.5$\,\,\pm \,\, 29.6$
$\checkmark$	–	Fine-tune	26.4$\,\,\pm \,\, 26.0$	**26**.**6**$\,\,\pm \,\, 31.1$	26.5$\,\,\pm \,\, 28.3$	29.1$\,\,\pm \,\, 24.3$	46.0$\,\,\pm \,\, 33.6$	36.5$\,\,\pm \,\, 29.1$
$\checkmark$	$\checkmark$	EWC [9]	27.5$\,\,\pm \,\, 25.6$	22.1$\,\,\pm \,\, 28.7$	25.2$\,\,\pm \,\, 27.1$	32.0$\,\,\pm \,\, 24.7$	44.9$\,\,\pm \,\, 33.9$	37.4$\,\,\pm \,\, 29.6$
$\checkmark$	$\checkmark$	L2SP [10]	**31**.**4**$\,\,\pm \,\, 27.3$	22.3$\,\,\pm \,\, 28.5$	27.5$\,\,\pm \,\, 28.2$	34.2$\,\,\pm \,\, 27.0$	41.2$\,\,\pm \,\, 35.6$	37.1$\,\,\pm \,\, 31.2$
$\checkmark$	$\checkmark$	BSS [22]	24.8$\,\,\pm \,\, 26.6$	18.3$\,\,\pm \,\, 27.6$	22.1$\,\,\pm \,\, 27.2$	27.9$\,\,\pm \,\, 25.5$	45.9$\,\,\pm \,\, 33.4$	35.5$\,\,\pm \,\, 30.4$
$\checkmark$	$\checkmark$	**ASFR**	30.5$\,\,\pm \,\, 27.6$	25.5$\,\,\pm \,\, 30.0$	**28**.**4**$\,\,\pm \,\, 29.0$	**37**.**0**$\,\,\pm \,\, 23.5$	**47**.**2**$\,\,\pm \,\, 32.1$	**41**.**3**$\,\,\pm \,\, 28.0$

Among the comparison methods, Baseline initializes the network without pretrained weights and, while capable in visible segments, struggles with occluding vessels. Fine-tune modifies the entire network based on pretrained weights, showing proficiency in detecting visible vessels but faltering with occluded ones. EWC and L2SP apply regularization to protect certain pretrained weights, enhancing the network’s segmentation ability for occluded vessels at the expense of reduced adaptability. BSS, a simpler classification network-based method, underperforms in the complexity of video segmentation tasks.

Our proposed ASFR method achieves an optimal balance, maintaining valuable information from the pretrained model while effectively adapting to the new task. Notably, for the XMem network, ASFR demonstrates superior localization of both visible and occluded vessels, evidenced by its higher average Dice scores.

### Qualitative evaluation

**Fig. 3:**
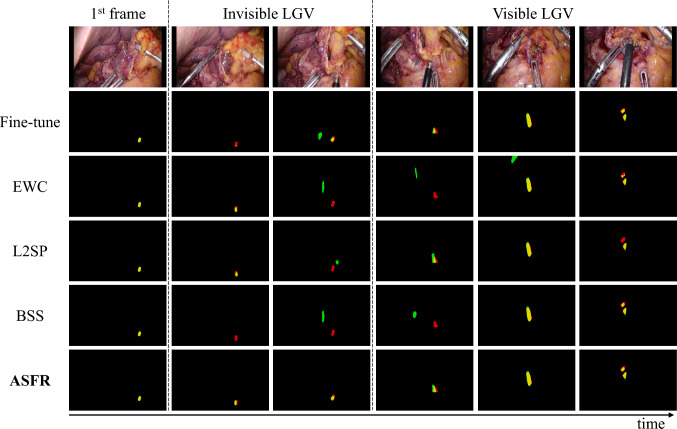
Comparison of qualitative segmentation results using different methods. Red indicates the annotated position of the LGV, green represents the network-predicted position of the LGV, and areas where both overlap appear in yellow. For invisible vessels, the ground truth annotations are based on estimations, therefore the boundaries of the ground truth may not be accurate

The qualitative outcomes of different methods are illustrated in Fig. 3. Our task involves predicting the positions of both invisible and visible LGV in subsequent frames, given annotations in the first frame. Among the provided examples, the most significant differences in predictions across methods occur during the crucial transition when the invisible vessels become visible. Compared to other methods, our proposed ASFR method consistently and accurately tracks the position of invisible vascular segments. This precision delivers superior segmentation results precisely at the critical juncture when the invisible vessels becomes visible.

Specifically, methods like Fine-tune and BSS struggle to effectively track the position of invisible vessels. EWC and L2SP manage to track vascular positions only over short durations. As the invisible vascular becomes discernible, the accuracy of predictions from these comparative methods markedly deteriorates, leading to subpar performance in the initial stages of visibility. However, once the vessel is clearly visible, all methods achieve intuitively acceptable prediction results.

### Experiments in the absence of initial frame annotations

**Fig. 4:**
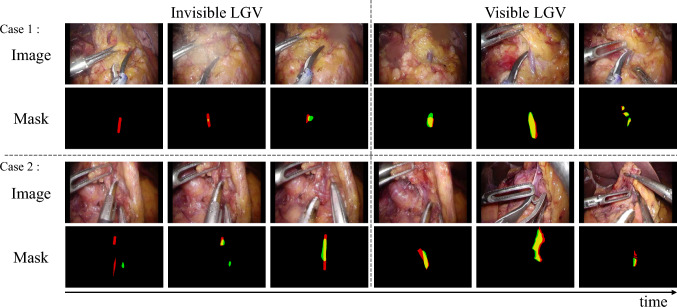
Predictions by the XMem network, fine-tuned with ASFR, without initial frame annotations, across two examples. The annotated LGV positions are marked in red, network predictions in green, and overlapping areas in yellow. The network fails to predict invisible vessel when obscured by dense adipose tissue. However, as the obscuring tissue becomes thinner, the network successfully detects the vessel, even when it remains not distinctly visible

**Table 2:** Comparison of Dice scores for transfer learning methods on the XMem network, evaluated without initial frame annotations. P indicates the use of pretrained weights, and R indicates the use of regularization. Highest and second highest results are highlighted in bold and underlined

P	R	Method	Invisible	Visible	Average
–	–	Baseline	3.8$\,\pm \, 12.6$	34.7$\,\pm \, 35.1$	16.8$\,\pm \, 29.1$
$\checkmark$	–	Fine-tune	10.4$\,\pm \, 20.4$	45.0 $\,\pm \, 35.1$	25.0$\,\pm \, 32.4$
$\checkmark$	$\checkmark$	EWC [9]	15.4$\,\pm \, 26.3$	36.9$\,\pm \, 34.0$	24.5$\,\pm \, 31.6$
$\checkmark$	$\checkmark$	L2SP [10]	**23**.**0**$\,\pm \, 29.3$	33.0$\,\pm \, 35.0$	27.2 $\,\pm \, 32.2$
$\checkmark$	$\checkmark$	BSS [22]	4.9$\,\pm \, 11.9$	43.7$\,\pm \, 34.8$	21.2$\,\pm \, 31.0$
$\checkmark$	$\checkmark$	**ASFR**	19.0 $\,\pm \, 24.1$	**45**.**6**$\,\pm \, 34.2$	**30**.**2**$\,\pm \, 31.6$

Although surgeons can utilize ICG [3] or Doppler ultrasound [4] to locate vessels obscured by adipose tissue, these techniques are not universally available in all laparoscopic surgeries. Additionally, in some cases, it is not feasible to provide initial annotations of invisible vessel in the first frame of the video. To evaluate our method’s performance under these conditions, we conducted a series of experiments.

The results are presented in Table 2 and Fig. 4. The XMem network, pretrained on large datasets and fine-tuned on the LGV dataset using our ASFR method, effectively identifies both visible and partially obscured LGV, even without initial frame annotations. Quantitatively, the accuracy in localizing visible vascular structures showed only minimal reduction compared to scenarios with annotated initial frames. In contrast, while the Fine-tune and BSS methods maintained consistent performance in segmenting obscured vessels, the EWC and L2SP methods exhibited significant performance declines.

Figure 4 notably demonstrates that certain methods can still identify sections of obscured vessels even without initial annotations, especially when the obstructive adipose tissue is relatively thin. These results highlight the capabilities of fine-tuned networks to effectively identify vascular structures during surgical procedures, emphasizing the robustness and adaptability of our proposed ASFR method in real clinical settings.

## Discussion

This study explores vascular localization in laparoscopic surgery, a field with great clinical potential but significant challenges due to often obscured views. Our approach focuses on fine-tuning pretrained models for medical image segmentation tasks, particularly valuable when working with limited training data. By leveraging rich feature representations from large-scale, non-medical datasets, we successfully adapted these models to the specialized requirements of medical image analysis.

Fine-tuning pretrained models proved highly effective, with our proposed ASFR method standing out. ASFR effectively balances knowledge retention and adaptability, enabling the detection of obscured vascular structures, especially when vessels are hidden by adipose tissue-a common issue in laparoscopic surgery. Our method demonstrated high accuracy even without initial frame annotations, which is a common challenge in clinical settings.

**Fig. 5:**
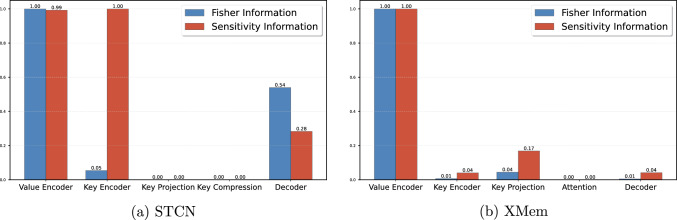
Comparison of module-specific Fisher information and sensitivity within STCN and XMem network. Notably, this analysis provides only a coarse-grained perspective, as significant differences exist within each module that are not captured here

We analysis the distribution of Fisher information and sensitivity across different modules within the networks. Figure 5 demonstrates the average values of the Fisher information and sensitivity across various network modules. To facilitate clear presentation and analysis, we ignore the internal structures of each module and the connections between modules, and only briefly discuss the overall average information weights of different modules and their potential implications.

Firstly, in both the STCN and XMem architectures, the value encoder-responsible for encoding current frame information-demonstrated the most significant impact on subsequent tasks. The value encoder contains crucial knowledge learned from the original task, as evidenced by its high responses in both Fisher information and sensitivity measures. Applying strong regularization constraints to the value encoder not only preserves its representational capabilities acquired during pretraining but also prevents overfitting due to excessive parameter adjustments when adapting to the new laparoscopic vascular segmentation task.

In contrast, the key encoder and key projection, which encode video context information, exhibited relatively lower Fisher information. This suggests they are less critical for preserving the model’s original capabilities. However, their higher sensitivity indicates potential overfitting risks without careful parameter adjustment, particularly given the limited laparoscopic video dataset. Therefore, it’s essential to adjust these modules cautiously to maintain generalization performance.

Moreover, the observed disparities in Fisher Information and Sensitivity between STCN and XMem architectures highlight ASFR’s adaptability across diverse network architectures. This flexibility is crucial for integrating ASFR into various video segmentation networks, ensuring its continued applicability as machine learning technologies evolve.

## Conclusion

This study introduces the ASFR method, a novel approach grounded in Heterogeneous Transfer Learning, tailored to enhance LGV detection in laparoscopic videos. Demonstrated through rigorous testing, ASFR shows a promising capability in identifying LGV with greater precision, affirming its potential as an effective solution for surgical image analysis challenges. This innovation in applying machine learning to medical image analysis paves the way for further advancements in laparoscopic surgery. Future work will explore improving ASFR’s performance without initial frame annotations and extending its application to other surgical areas, potentially broadening its clinical impact. This approach promises to refine surgical procedures by improving the precision and reliability of intraoperative image analysis, thereby contributing to enhanced patient outcomes and surgical efficiency.

## Supplementary information

The supplementary materials include two videos (6 and 7 min) showcasing experimental results on our in-house dataset and a PDF providing additional details about the dataset and the videos.

## Supplementary Information

Below is the link to the electronic supplementary material.

Supplementary file 1 (mp4 101437 KB)

Supplementary file 2 (mp4 100756 KB)

Supplementary file 3 (pdf 1801 KB)
