# Supplementary material for: Adaptive sensitivity-fisher regularization for heterogeneous transfer learning of vascular segmentation in laparoscopic videos
Source: Int J Comput Assist Radiol Surg. 2025 Jun 6;20(7):1503–11. doi: 10.1007/s11548-025-03404-2 (PMC12226648; doi:10.1007/s11548-025-03404-2)
Supplement: Supplementary file 3 — (pdf 1801 KB) [file 11548_2025_3404_MOESM3_ESM.pdf]

Adaptive Sensitivity-Fisher Regularization for Heterogeneous Transfer Learning of Vascular Segmentation in Laparoscopic Videos -Supplementary Material-

Table 1 Dataset Composition for Training, Validation, and Testing.

**Dataset Partition**	**Set**	**Cases**	**Labelled Frames**	
			**Invisible**	**Visible**
**Training**	Invisible Vessel Only	7	1923	0
	Visible Vessel Only	8	0	897
	Visibility Transition of Vessels (Invisible to Visible)	2	235	158
**Validation**	Visibility Transition of Vessels (Invisible to Visible)	2	401	186
**Testing**	Visibility Transition of Vessels (Invisible to Visible)	4	885	340

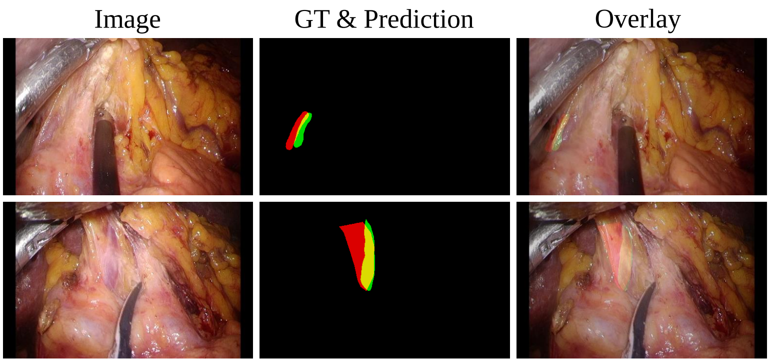

Fig. 1 Visualization of laparoscopic images with corresponding vascular annotations. Red represents the ground truth (GT), and green represents the network predictions. Note that defining clear boundaries for vascular structures is challenging; therefore, there is a lack of a definitive gold standard for evaluation.

Table 2 Brief notes on the supplementary material video 1.

**Time**	**Frame**	**Notes**
0:00	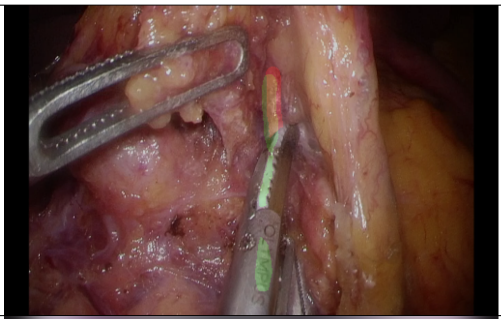	The vessels are obscured by tissues and surgical instruments.
2:35	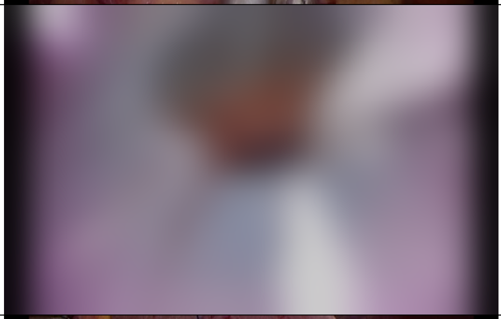	The camera exits the body (this part of the frame has been blurred to protect privacy).
3:20	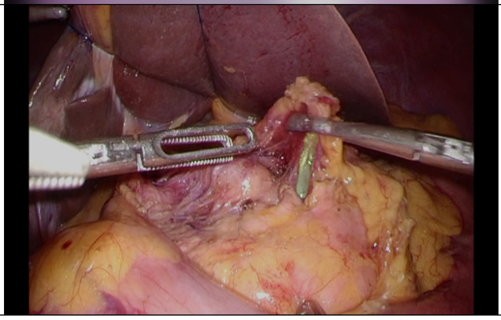	The camera returns inside the body and continues to segment the vessels.

Table 3 Brief notes on the supplementary material video 1.

Time	Frame	Notes
0:00	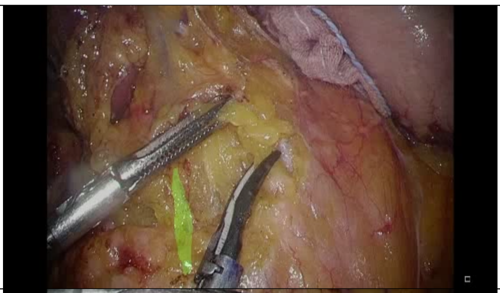	The LGV vessel below the visible vessels is the target.
3:20	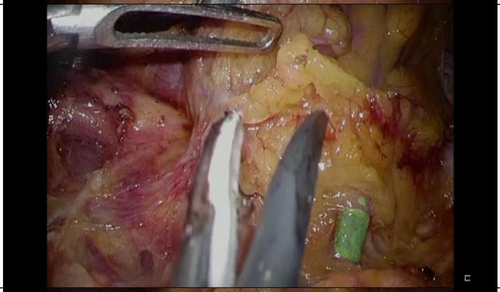	The target vessel is continuously segmented without interference from the visible vessels.
6:26	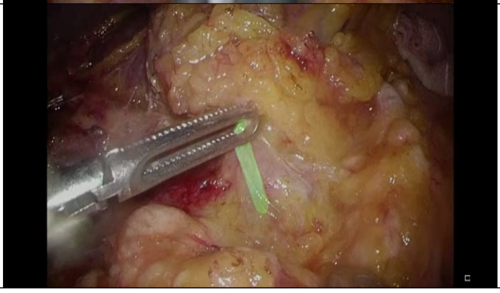	Finally, the surgical tool grasps the target vessel.
